# Differential Nutrition-Health Properties of *Ocimum basilicum* Leaf and Stem Extracts

**DOI:** 10.3390/foods11121699

**Published:** 2022-06-09

**Authors:** Aicha Bensaid, Frederic Boudard, Adrien Servent, Sylvie Morel, Karine Portet, Caroline Guzman, Manon Vitou, Florence Bichon, Patrick Poucheret

**Affiliations:** 1 Qualisud, University Montpellier, Avignon Université, CIRAD, Institut Agro, IRD, Université de La Réunion, 34000 Montpellier, France; aichatasnime826@gmail.com (A.B.); frederic.boudard@umontpellier.fr (F.B.); adrien.servent@cirad.fr (A.S.); karine.portet@umontpellier.fr (K.P.); caroline.guzman@umontpellier.fr (C.G.); florence.bichon@umontpellier.fr (F.B.); 2 Laboratoire de Botanique, Phytochimie et Mycologie, CEFE, CNRS-Université de Montpellier-Université Paul-Valéry Montpellier-EPHE-IRD, 34000 Montpellier, France; sylvie.morel@umontpellier.fr (S.M.); manon.vitou@umontpellier.fr (M.V.)

**Keywords:** *Ocimum basilicum*, stems, leaves, nutrition, health, anti-oxidant, anti-inflammatory, antispasmodic

## Abstract

(1) Background: *Ocimum basilicum* L. is an aromatic medicinal plant of the Lamiaceae family known as sweet basil. It is used in traditional medicine for its beneficial effects on gastrointestinal disorders, inflammation, immune system, pyrexia or cancer among others. *Ocimum basilicum* (OB) leaf extracts contain many phytochemicals bearing the plant health effects but no reports is available on the potential bioactivity of stem extracts. Our investigation aimed at assessing the differential biological activity between basil leaf and stem to promote this co-product valorization. (2) Method: For this purpose we explored phytochemical composition of both parts of the plant. Antioxidant activity was evaluated through total polyphenol content measure, DPPH and ORAC tests. Anti-inflammatory markers on stimulated macrophages, including NO (nitric oxide), TNFa (tumor necrosis factor alpha), IL-6 (interleukin 6), MCP1 (monocyte attractant protein 1) and PGE-2 (prostaglandin E2), were evaluated. In addition, we investigated OB effects on jejunum smooth muscle contractility. (3) Results: OB extracts from leaves and stems demonstrated a different biological activity profile at the level of both antioxidant, anti-inflammatory and smooth muscle relaxation effects. (4) Conclusion: Taken together our results suggest that *Ocimum basilicum* extracts from co-product stems, in addition to leaves, may be of interest at the nutrition-health level with specific therapeutic potential.

## 1. Introduction

*Ocimum basilicum* L. is an aromatic medicinal plant belonging to the Lamiaceae family known as sweet basil and is also called the king of the herbs [1]. It is an annual plant widely distributed and cultivated around the world. This herb genus, encompassing over 100 aromatic species, is native to Asia, India, Africa, South America. Basil has found a wide range of applications in various fields such as in the traditional medicine, pharmaceutical, cosmetical, nutraceutical and food industries [2,3]. Its aromatic profile makes it a very common spice for meal preparation in various cultures to provide aroma and flavor [4]. The most used parts of the plant are the leaves and the seeds. Leaves may be used for cooking, production of essential oils and as a component of various beverages. Seeds are integrated as a functional ingredient in food and non-food product processing. Conversely, stems are seldom reported for their use. They may find application in food flavoring [5,6] and one reference indicates a traditional medicine usage [1].

The health effects of *Ocimum basilicum* L. (OB) are recognized and described in the scientific literature. It contains over 200 bioactive phytochemical compounds from various families. In addition to macronutrients, carbohydrate, lipids, proteins and mucilages, basil contains micronutrients including vitamins, minerals, and secondary metabolites such as polyphenols, flavonoids, flavones. It also contains essential oil with terpenic compounds, monoterpene and sesquiterpenes, i.e., linalool, limonene, geraniol, caryophyllene and alpha-cadinol [7]. Basil secondary metabolites and more specifically phenolic bioactive compounds have been explored. Among the main flavonoids molecules found in basil, quercetin and rutin are the most represented, along with catechin, kaempferol and luteolin. The major phenolic compounds found in OB are reported to be rosmarinic acid followed by caffeic acid derivatives. The main derivatives are usually chicoric, caftaric, caffeic, coumaric, gallic and chlorogenic acids [8].

Health benefits attributed to *Ocimum basilicum* L. rely on its content of secondary metabolites, i.e., phenolic and flavonoid compounds. OB leaf bioactivities are the most frequently described in the scientific literature and in traditional medicine [1,9]. The therapeutic potentials of leaf extracts appear quite diverse. They are used for headache, respiratory tract inflammation (sore throat, laryngitis, bronchitis), IBD (Intestinal Bowel Disease), kidney dysfunction, and as anti-pyretic and anti-cancer agents, among others applications [10,11,12]. In addition, OB extracts appear to generate beneficial pharmacological effects as antioxidant, anti-inflammatory, analgesic and antispasmodic agents. They act on metabolic and immune diseases with chronic inflammation such as metabolic syndrome and intestinal bowel diseases [13,14,15].

The bioactivities of plant extracts are known to be influenced by the accession type, growing conditions, and harvest period during the year [16,17]. Indeed, geography, pedology and season modulate the quality and relative quantities of phytochemical compounds, and therefore the optimal yield of active principles, thereby altering the health effect potential [18]. In the same perspective, as reported by Asgari-Lajayer et al., elicitation (such as decontaminated sewage sludge intrants) may also selectively induce plant metabolic pathways. This would generate specific phytochemical profile with intended bioactivities [19]. In addition, the choice of the subparts sampled from the plant may be a determinant of the bioactivity profile. Each part of a vegetal may have a specific qualitative and quantitative chemical profile due to differential distribution of phytochemicals as a function of organ role and proper metabolic pathways [20,21]. In this context, each part of a vegetable may bear differential bioactivity, putatively making it possible to address specific pharmacological objectives and to valorize plant subparts otherwise considered to be low-value co-products.

Most studies on basil species focused on leaves and essential oils. A few reports explored stems antioxidant and/or anti-inflammatory properties of *Ocimum sanctum* L. and *Ocimum Americanum* L. respectively [22,23,24]. Regarding anti-spasmodic properties, they were studied on ileum with *Ocimum gratissimum* L. and *Ocimum selloi* L. essential oils but not on specific plant subpart [25,26,27,28]. Hence, no investigation explored these combined bioactivities on OB in order to provide elements of comparison between the various species health effects.

Therefore the objective of the present study was to explore the differential phytochemical, antioxidant, anti-inflammatory and antispasmodic profiles of *Ocimum basilicum* L. leaf and stem extracts. These investigations aimed at better understanding the various bioactive potentials of OB and to unravel the nutrition-health potential of the stem co-product. To our knowledge, it is the first time that such exploration has been undertaken on *Ocimum basilicum* L.

## 2. Materials and Methods

### 2.1. Plant Material

The aerial parts of *Ocimum basilicum* were collected in February 2020 in Kenya from a single local producer. They were imported by a French aromatic plants wholesaler at full commercial maturity (4 months of growth). Basil identification was performed and confirmed by a botanist, Sylvie Morel. A specimen was deposited at the laboratory of Botany and Mycology of the Faculty of Pharmaceutical Sciences of the University of Montpellier.

### 2.2. Preparation of Crude Extracts

Basil aerial parts (leaves, stems) were air-dried and grounded to powder in an herbal mill (Thermomix). Four aqueous and ethanolic extracts were prepared from basil stems and leaves. A sample of 50 g of leaf or 50 g of stem powders were extracted five times using 200 mL H_2_O HPLC (high pressure liquid chromatography) grade or 200 mL absolute ethanol. An ultrasound exposure of 45 KHz for 10 min was applied. The extracts were then filtrated. Following these steps, extracts were dried under high pressure for solvent removal after being weighed to determine the extraction yield. Extracts were stored at 20 °C for further analysis.

### 2.3. Fingerprint of Polyphenols Extracts

The basil extracts were analyzed using liquid chromatography coupled with a mass spectrometer Acquity UPLC (Waters, Milford, MA, USA). Compounds were analyzed based on their retention times, their UV–Vis spectra, and their mass spectra. Mass spectrum was acquired using a Synapt G2-S (Waters, Milford, MA, USA) set at ESI-ionization, for a range of mass of 50–1600 Da, with a source at 140 °C, a capillary tension of 3 kV and a desolvation temperature of 450 °C with the same chromatographic parameters. Basil extracts were prepared in distilled water and methanol (30 mg/mL), and 20 µL was injected. The instrument was equipped with a column C_18_ ACE 250 mm × 4.6 mm × 5 µm (Advanced Chromatography Technologies Ltd., Aberdeen, Scotland). DAD (Diode Array Detection) was set at 280, 330, and 380 nm. Mobile phases were 1% formic acid in pure water as phase A and acetonitrile as phase B. Flow was set at 0.7 mL min^−1^ and at 30 °C. Gradient was fixed at 98% of A and 2% of B (at initial stage), stabilized at 2% B for 1 min, increasing at 20% of B from 1 to 3 min, to 40% B from 3 to 6 min, to 60% B from 7 to 8 min, to 80% B from 8 to 9 min, to 100% B from 9 to 11 min, returned to initial condition (2% B) in 3 min and maintained for 3 min.

### 2.4. Antioxidant Activity

#### 2.4.1. TPC (Total Phenolic Content) Test

Total polyphenols assay was performed with Folin-Ciocalteu reagent according to the method of Morel et al., 2018 [29]. Extracts of OB and of rosemary were prepared in DMSO and tested at a concentration of 1 mg/mL. A calibration curve was generated on a concentration range of 1.56 to 75 μg/mL) of gallic acid. In a 96-well plate, 50 μL of extract or 50 μL of gallic acid, and 50 μL of distilled water were distributed in triplicate. Then 50 μL of 10% Folin Ciocalteu reagent and 50 μL of sodium carbonate solution (1M) were added to all wells. The absorbance was measured on a microplate reader (Molecular Devices) at a wavelength of 650 nm. Results are expressed as milligrams of gallic acid equivalents (GAE) per gram of OB plant leaf extract.

#### 2.4.2. DPPH (2,2-Diphenyl-1-picrylhydrazyl) Scavenging Activity

Antioxidant activity was evaluated using the DPPH assay according to the method of Morel [29]. Extracts were solubilized in DMSO (4 mg/mL) before being diluted in absolute ethanol to reach a concentration of 1 mg/mL. A standard curve of Trolox was performed (75, 50, 25, 12.5 µM). Ethanol was used as blank, ethanolic extract of *Rosmarinus officinalis* (0.2 mg/mL) and chlorogenic acid (0.01 mg/mL) were used as positive controls. In a 96-well plate, 100 µL of positive control or extract were placed in each well. The test was performed in triplicate for each extract. 75 µL of absolute ethanol and 25 µL of extemporaneously prepared DPPH solution (0.4 mg/mL) were introduced into each well. The plate was incubated for 30 min at room temperature and protected from light. The absorbance was read at 550 nm with a microplate reader (MDS Inc., Toronto, ON, Canada). Results are expressed as the mean plus or minus standard deviation of three independent experiments and are expressed as Trolox equivalents (TE µmoles per gram of dry extract). Results are also expressed as percentage of inhibition (% inhibition).

#### 2.4.3. ORAC (Oxygen Radical Absorbance Capacity) Assay

The ORAC assays were performed in 96-well polypropylene plates as previously described [29]. Samples were solubilized in DMSO at a concentration of 1 mg/mL before being diluted to 25 µg/mL using phosphate buffer at pH 7.4. On the 96-well microplate, 20 µL of Trolox solutions at 0.6, 25, 12.5, 25, 50 and 75 µM as standard curve, or chlorogenic acid (0.01 mg/mL), or ethanolic extract of rosemary (12.5 µg/mL) as a positive control, or the extracts at a concentration of 25 µg/mL, were applied. Then, 100 µL of phosphate buffer and 100 µL of extemporaneously prepared fluorescein solution (0.1 µM in phosphate buffer) were added. The microplate was incubated at 37 °C for 10 min with shaking. The reaction was initiated with 50 µL of AAPH (2,2′-azobis(2-amidino-propane) dihydrochloride). Fluorescence was recorded at an excitation wavelength of 485 nm and an emission wavelength of 535 nm, for 70 min using a Tristar LB 941 microplate reader. Final ORAC values were calculated using a regression equation between Trolox concentration and area under the curve of decreasing fluorescein. Data are expressed as µmoles of Trolox equivalents per gram of dry extract.

### 2.5. Anti-Inflammatory Activity

#### 2.5.1. Macrophage Culture

The macrophage cell line J774.A1 (ATCC, TIB67) was obtained from LGC Standards. Cells were cultured in RPMI 1640 GlutaMAX^®^ medium supplemented with streptomycin (100 µg/mL) and penicillin (100 units/mL), 10% inactivated fetal calf serum complete RPMI medium), cells were incubated in an incubator at 37 °C, 5% CO_2_, 95% humidity.

#### 2.5.2. Cell Viability Assay

To test cytotoxicity, 6 × 10^5^ cells/well were seeded in a 96-well culture plate in complete RPMI medium and incubated at 37 °C with different concentrations of extracts (50, 75, 100, 150 and 200 μg/mL) for 20 h. After incubation, 20 μL/well of (3-(4,5-dimethylthiazol-2-yl)-5-(3-carboxymethoxyphenyl)-2-(4-sulfophenyl)-2H-tetrazolium), MTS, mixed with an electron coupling reagent, PMS in HBSS, was added. The plate was incubated for an additional 4 h and the absorbance at 490 nm was measured in a microplate reader (Molecular Devices) as previously described. The cell viability was calculated according to the following formula:

cell viability % = absorbance of samples × 100/absorbance of control.

#### 2.5.3. Determination of Nitrites (NO)

Extracts at the same concentrations were used as a pretreatment for the determination of the amount of nitrite produced. The presence of nitrite, a stable oxidized product of nitric oxide, was determined in the cell culture media as previously described [30]. Briefly, 100 μL of supernatant were combined with 100 μL of Griess reagent in a 96-well plate, incubated 10 min at room temperature. Nitrite concentration was determined by measuring absorbance at 550 nm and using a NaNO_2_ standard curve (1.56 to 100 μM).

Results were expressed as percentage of inhibition values.

#### 2.5.4. TNF-α (Tumor Necrosis Factor Alpha) Assay

The tumor necrosis factor alpha (TNF-alpha) assay was performed according to the instructions contained in the kit-ELISA (TNF alpha Mouse Uncoated ELISA kit; Thermo Fisher Scientific, Waltham, MA, USA). After pretreatment with the different concentrations of OB extracts for 4 h, the cells were stimulated with LPS (lipopolysaccharide) 100 ng/mL (*E. coli*, 555B5) and mouse INFγ 10 ng/mL for 3 h. TNF-α release in cell supernatants was tested by sandwich enzyme-linked immunosorbent ELISA (enzyme linked immunosorbent assay) assay.

#### 2.5.5. IL-6 (Interleukin 6) Assay

IL-6 production by J774 cells was determined with the IL-6 ELISA-kit (Mouse IL6 ELISA; Thermo Fisher Scientific, Waltham, MA, USA) after pretreatment with OB extracts at a determined concentration range (50, 75, 100, 150 and 200 μg/mL) for 4 h. The cells were stimulated with 100 ng/mL LPS (*E. coli*, 555B5) and 10 ng/mL mouse INFγ for 18 h. IL-6 release in cell supernatants was tested according to the ELISA Kit instructions. The results for IL-6 as well as for all other pro-inflammatory cytokines are expressed as percentage of inhibition values.

#### 2.5.6. Prostaglandin Assay

The determination of prostaglandin E2 was performed by the competitive enzyme-linked immunosorbent assay (ELISA) on culture supernatants after pretreatment and subsequent activation of the cells with LPS/IFNγ using the commercial Cayman PGE2 ELISA Kit Monoclonal.

#### 2.5.7. MCP-1 (Monocyte Chemoattractant Protein-1) Assay

Using the ELISA kit (Mouse CCL2 (MCP-1) (Thermo Fisher Scientific, Waltham, MA, USA) MCP-1 was detected in the cell culture supernatant 18 h after activation with LPS/IFNγ. The supernatant was diluted 1:10 in EIA buffer to obtain a concentration of MCP-1 within the calibration range.

### 2.6. Antispasmodic Activity

#### 2.6.1. Antispasmodic Model

These experiments were carried out in accordance with the Declaration of Helsinki and with the Guide for the Care and Use of Laboratory Animals as adopted and promulgated by the US National Institutes of Health. Our laboratory practice and protocols were approved on 8 February 2012, by the legal institution “Comité d’Ethique pour l’Expérimentation Animale Languedoc–Roussillon” with the bioethical approval code CEEA-LR-13015 for the University of Montpellier.

##### Animal Model and Isolated Jejunum Preparation

Basil antispasmodic activity on jejunum smooth muscle was performed with OB aqueous extract (since ethanol extracts compromised organ integrity and response). Male Wistar rats weighing (336–428 g) were purchased from Janvier^®^. Animals were maintained under stable environmental conditions (temperature 22 ± 2 °C), with a light/dark cycle (12/12 h).

Rats were anesthetized and euthanized by exsanguination. Jejunum was carefully removed and placed in Tyrod buffer whose composition was: NaCl (137 mmol/L), KCl (2.7 mmol/L), MgCl_2_ (1.1 mmol/L), CaCl_2_ (1.8 mmol/L), NaHCO_3_ (11.9 mmol/L), glucose (5.5 mmol/L). pH was 7.4. Jejunum was then washed, released from mesenteric attachments and cut into small segments of 10 mm in length. The fragments were then mounted on organ holders and placed in 9 mL double-walled isolated isolated organ tanks (EMKA) filled with Tyrode buffer. The physiological medium was maintained at 32 °C and oxygenated by a mixture of O_2_/CO_2_ (95%:5%). For the assembly of the jejunum fragments, the lower part of the organ holder was immobilized in the isolated organ tank and the upper part was connected to an isometric tension sensor. The signal supplied by the sensor was analyzed using the computer system Mac-Lab V3.6 (Apple, Cupertino, CA, USA).

##### Spasmolytic Activity

At the start of each experiment, jejunum segments were subjected to a basal tension of 1 g. After an equilibration period of 40 min during which washes were performed every 10 min, stimulation of the jejunum fragments was induced by adding acetylcholine (10^−5^ M) into the tank. After acetylcholine contraction, a second equilibration period of 40 min was performed during which washings were carried out every 10 min. After this equilibration period, the jejunum fragments were subjected to a contraction induced either by BaCl_2_ (4 × 10^−4^ M) or methacholine (3 × 10^−6^ M) for 5 min. After another 40 min equilibration period in Tyrod buffer, different concentrations of *Ocimum basilicum* leaf or stem aqueous extracts (1 mg/mL, 3 mg/mL, 6 mg/mL, 10 mg/mL) were added for a 10 min pre-treatment. A second contraction with either BaCl_2_ (4 × 10^−4^ moL) or methacholine (3 × 10^−6^ moL) was then performed.

### 2.7. Statistical Analysis

Values are given as mean ± SD (standard deviation). All statistical analysis were performed on XLSTAT Software version 2019.4.1 and using one-way ANOVA followed by post hoc Tukey test. Differences were considered significant when *p* < 0.05.

## 3. Results

### 3.1. Phytochemical Analysis

**Figure 1:**
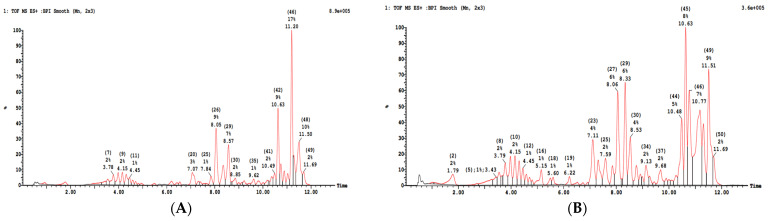
Chromatogram of ocimum basilicum ethanolic leaf extracts (**A**) and ethanolic stem extracts of ocimum basilicum (**B**) (M-H^+^).

As summarized in Figure 1, Table 1 and Table 2, the phenolic profiles of leaf and stem ethanolic extracts obtained by liquid chromatography were different. Similar results were obtained for aqueous extracts. Ethanolic and aqueous extracts for both leaves and stems were different, as was to be expected, considering the difference of extraction solvant. Phytochemical profiles demonstrated variations at both qualitative and quantitative levels. Chromatogram revealed the presence of various compounds belonging mainly to the phenolic acids, the flavonoids, organic acids and fatty acids families in accordance with the literature [31]. Phytochemicals were identified according to their chromatographic parameters (retention time, molecular ionization, etc.), internal database correspondence, and literature information. The main compounds present in leaf extracts (combined from aqueous and ethanolic) were ferroyl-tartaric acid (11%), stearic acid (9%), salvigenin (5%), medioresinol (4%), rutin (2%), and gallo-catechin (1%). In the stem extracts (combined from aqueous and ethanolic), the main compounds were vicenin (8%), rosmarinic acid (7%), stearic acid (6%), salvigenin (6%) and salvianolic acid (6%). These data indicated differential phytochemical profiles between leaves and stems associated with the specific relative ratio of bioactive compounds in each of the two matrixes. Such variations may be associated with specific biological properties.

### 3.2. Polyphenols

**Figure 2:**
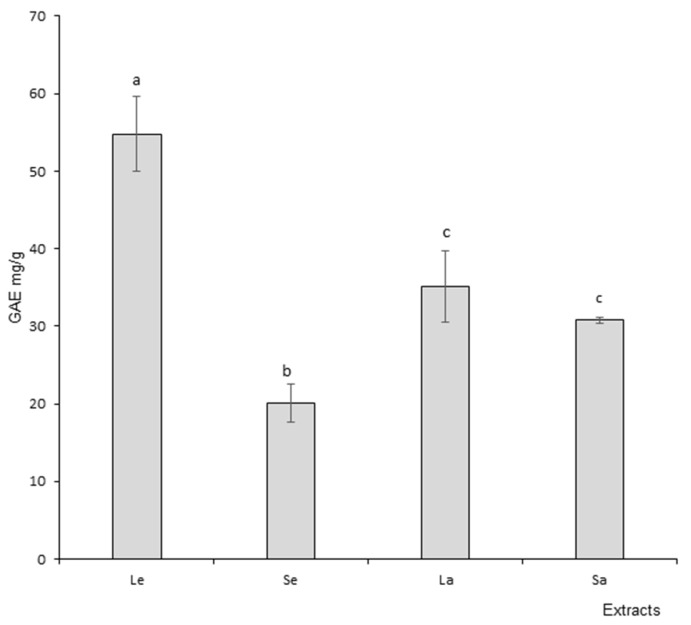
Total polyphenol content (TPC) of Ocimum basilicum extracts. Le: leaf ethanol extracts; Se: stem ethanol extracts; La: leaf aqueous extracts; Sa: stem aqueous extracts. The values are expressed as mean ± SD (*n* = 3). The dissimilar letters on the graph represent significant differences (*p* < 0.05) according to one-way ANOVA followed by post hoc Tukey test. Error bars indicate ± SD (standard deviation).

Results obtained with the TPC assay are presented in Figure 2, which presents phenolic content expressed as GAE (Gallic Acid Equivalent). The TPC results indicated a significantly higher polyphenol content in leaf ethanol extract when compared to stem ethanol extract. Regarding aqueous extracts, the difference between leaves and stems was not significant; also, the absolute value was higher in leaves (35.14 mg/g GAE) than in stems (30.75 mg/g GAE). In addition, leaf ethanol extracts demonstrated higher polyphenol content when compared to aqueous leaf extracts. The opposite result was recorded regarding stems.

#### DPPH and ORAC Assay Results

**Figure 3:**
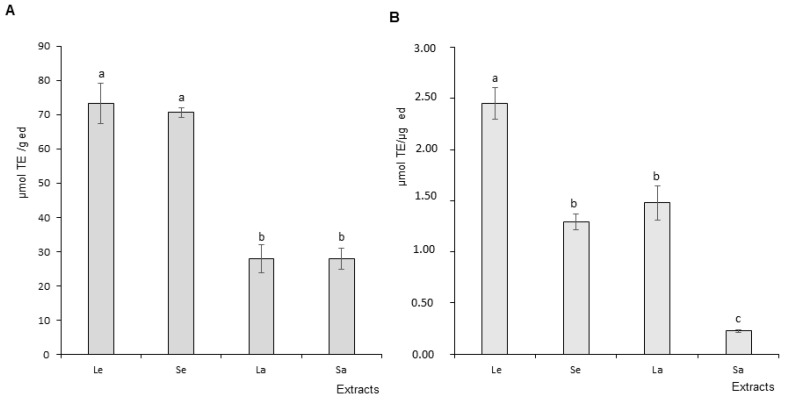
Antioxidant capacity of Ocimum basilicum extracts. (**A**) DPPH assay results (1,1-diphenyl-2-picrylhydrazyl), (**B**) ORAC assay results (oxygen radical absorbance capacity). Le: leaf ethanol extracts; Se: stem ethanol extracts; La: leaf aqueous extracts; Sa: stem aqueous extracts. The values are expressed as mean ± SD (*n* = 3). The dissimilar letters on the graph represent significant differences (*p* < 0.05) according to one-way ANOVA followed by post hoc Tukey test. Error bars indicate ± SD (standard deviation).

As expected, all Ocimum basilicum extracts presented antioxidant activity to various degrees in both assays. Figure 3A presents the DPPH assay results expressed in TE (Trolox equivalent). Leaf and stem ethanol extracts demonstrated equivalent radical scavenging potential. Similarly, leaf and stem aqueous extracts antioxidant activity were not statistically different. It should be mentioned that ethanol extracts were more active that aqueous extracts. Figure 3B presents the ORAC assay results. Leaf ethanol extract antioxidant capacity was statistically higher than stem ethanol extracts. Similarly, the same difference was recorded between leaf and stem aqueous extracts, with leaves bearing a more elevated activity than stems.

Overall, ethanol extracts tended to demonstrate higher bioactivity than aqueous extracts. Therefore, anti-inflammatory assays on culture cells were only performed on leaf and stem ethanol extracts.

### 3.3. Anti-Inflammatory Activity

#### 3.3.1. Cell Viability

Cell exposure to Ocimum basilicum extracts did not alter macrophage viability, thereby making it possible to explore anti-inflammatory activity without adverse influence.

#### 3.3.2. Nitric Oxide (NO) Production and Scavenging

##### Nitric Oxide (NO) Production

**Figure 4:**
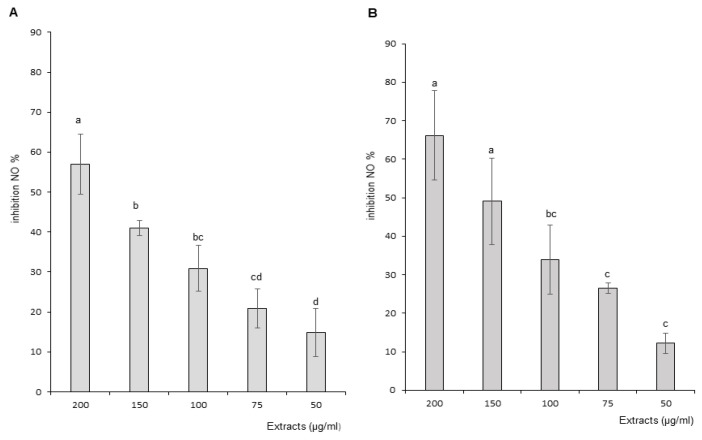
Effect of Ocimum basilicum extracts on nitric oxide (NO) production. (**A**) Leaf ethanol extract results, (**B**) stem ethanol extract results. The values are expressed as mean ± SD (*n* = 3). The dissimilar letters on the graph represent significant differences (*p* < 0.05) according to one-way ANOVA followed by post hoc Tukey test. Error bars indicate ± SD (standard deviation).

The effects of OB extracts on nitric oxide (NO) production are presented in Figure 4A,B. The graph represents the level of inhibition of NO production as a function of increasing concentrations of OB extracts (50, 75, 100, 150 and 200 μg/mL). Leaf and stem ethanol extracts demonstrated a concentration-dependent inhibition of NO production by stimulated macrophage cells with statistically significant differences between at least three concentrations. Inhibition potential ranged for leaves and stems respectively from 14.81 to 56.95% and from 12.17 to 66.18%. Therefore leaf and stem extracts decreased stimulated macrophage production of the pro-inflammatory free radical. No statistically significant differences were recorded between NO production inhibitory potential of leaves and stems.

##### Nitric Oxide (NO) Scavenging

**Figure 5:**
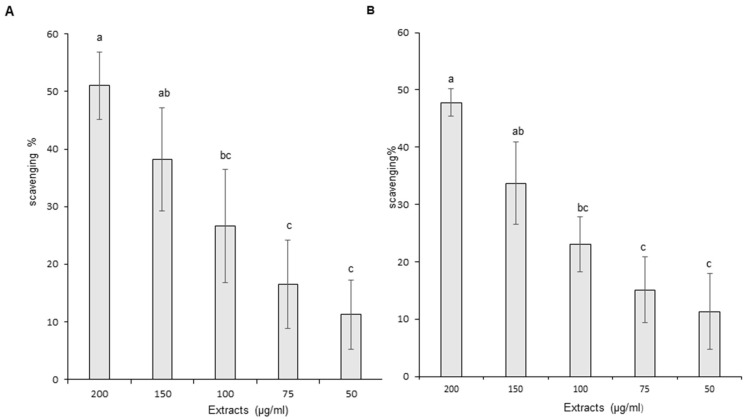
Scavenging capacity of Ocimum basilicum extracts on nitric oxide (NO). (**A**) Leaf ethanol extract results, (**B**) stem ethanol extract results. The values are expressed as mean ± SD (*n* = 3). The dissimilar letters on the graph represent significant differences (*p* < 0.05) according to one-way ANOVA followed by post hoc Tukey test. Error bars indicate ± SD (standard deviation).

Nitric oxide (NO) scavenging results are presented on Figure 5A,B. The graph presents the level of NO scavenging potential at increasing concentrations of OB leaf and stem ethanol extracts. Both types of extract demonstrated statistically significant concentration-dependent differences in NO scavenging capacity between at least three concentrations. Scavenging potential ranged for leaves and stems, respectively, from 11.26 to 51.02% and from 11.35 to 47.79%. Therefore, leaf and stem extracts were able to partially quench the pro-inflammatory free radicals. No statistically significant difference was recorded between the NO scavenging effects of leaves and stems.

#### 3.3.3. Tumor Necrosis Factor Alpha (TNF-α)

**Figure 6:**
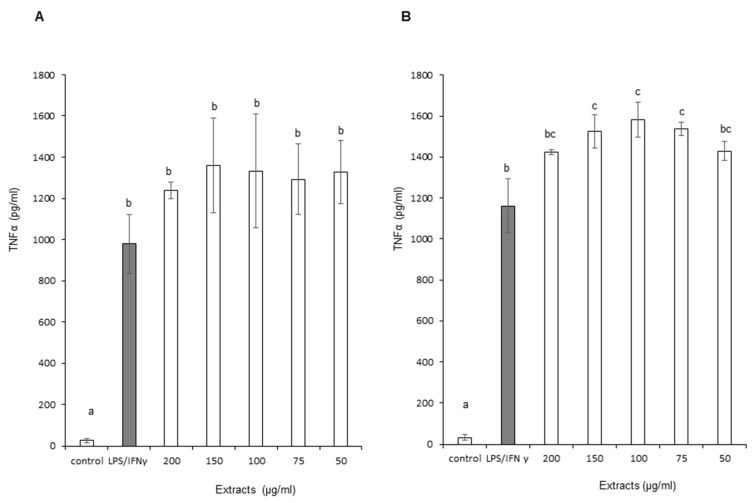
Effect of Ocimum basilicum extracts on Tumor Necrosis Factor alpha (TNF-α) production. (**A**) Leaf ethanol extract results, (**B**) stem ethanol extract results. The values are expressed as mean ± SD (*n* = 3). The dissimilar letters on the graph represent significant differences (*p* < 0.05) according to one-way ANOVA followed by post hoc Tukey test. Error bars indicate ± SD (standard deviation).

Figure 6A,B show the impact of OB extracts on TNF-α production by stimulated macrophage cells. The graph represents the level of TNF-α production as a function of increasing concentrations of OB extracts. Leaf and stem ethanol extracts did not demonstrate a statistically significant reduction in TNF-α production. Moreover, a slight statistically significant increase in TNF-α production was recorded with stem extracts. A similar phenomenon was observed with leaf extracts, even though it did not reach statistical significance due to larger errors to the mean. Therefore, leaf and stem extracts did not positively influence TNF-α with regard to anti-inflammatory effect.

#### 3.3.4. Interleukin 6 (IL-6)

**Figure 7:**
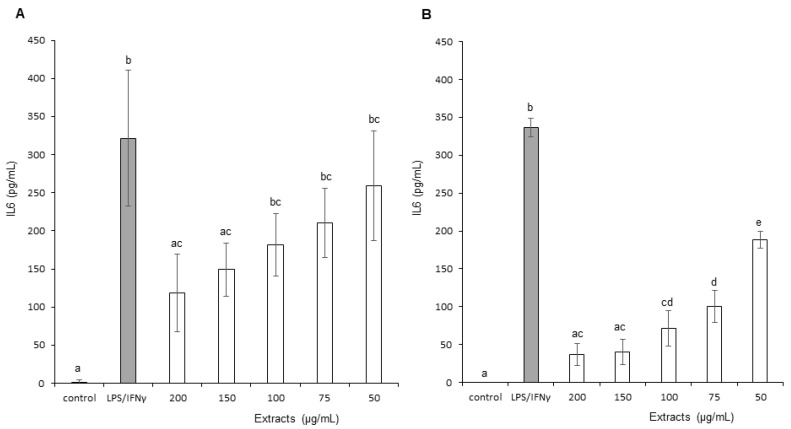
Effect of Ocimum basilicum extracts on Interleukin 6 (IL-6) production. (**A**) Leaf ethanol extract results, (**B**) stem ethanol extracts results. The values are expressed as mean ± SD (*n* = 3). The dissimilar letters on the graph represent significant differences (*p* < 0.05) according to one-way ANOVA followed by post hoc Tukey test. Error bars indicate ± SD (standard deviation).

The effects of OB extracts on Interleukin 6 (IL-6) production are presented in Figure 7A,B. The graph presents the level of IL-6 production as a function of increasing concentrations of OB extracts. Leaf and stem ethanol extracts demonstrated a concentration-dependent reduction in IL-6 production by stimulated macrophage cells. In addition, stem extracts decreased IL-6 levels more significantly than leaf extracts, with maximum reductions of, respectively, 88.87% and 62.43%. Therefore, stem extracts demonstrated a superior effect on IL-6 inflammation marker.

#### 3.3.5. Monocyte Chemoattractant Protein-1 (MCP-1)

**Figure 8:**
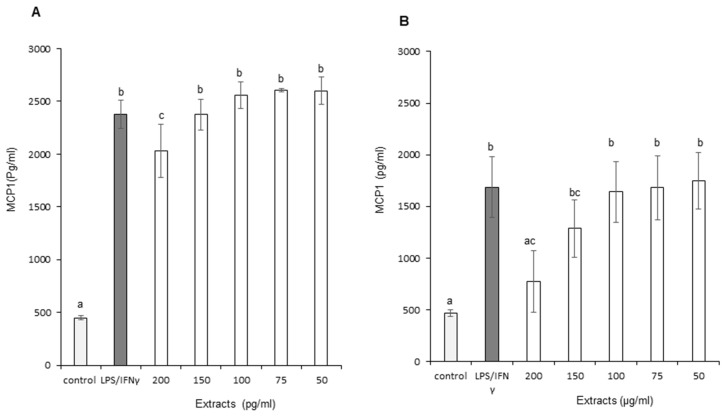
Effect of Ocimum basilicum extracts on Monocyte Chemoattractant Protein-1 (MCP-1) production. (**A**) Leaf ethanol extract results, (**B**) stem ethanol extracts results. The values are expressed as mean ± SD (*n* = 3). The dissimilar letters on the graph represent significant differences (*p* < 0.05) according to one-way ANOVA followed by post hoc Tukey test. Error bars indicate ± SD (standard deviation).

The effects of OB extracts on Monocyte Chemoattractant Protein-1 (MCP-1) production are presented in Figure 8A,B. The graph represents the level of MCP-1 production as a function of increasing concentrations of OB extracts. Leaf extracts did not generate a reduction in MCP-1 production except at the highest dose (200 μg/mL), with an average decrease of 14.73% for this concentration. Conversely, stems reduced MCP-1 production in a partially concentration-dependent way at 150 and 200 µ/mL of OB extracts, with an average decrease of, respectively, 23.92% and 55.28%. In addition, therefore, stem extracts demonstrated a superior effect on MCP-1 inflammation marker.

#### 3.3.6. Prostaglandin E2 (PGE-2)

**Figure 9:**
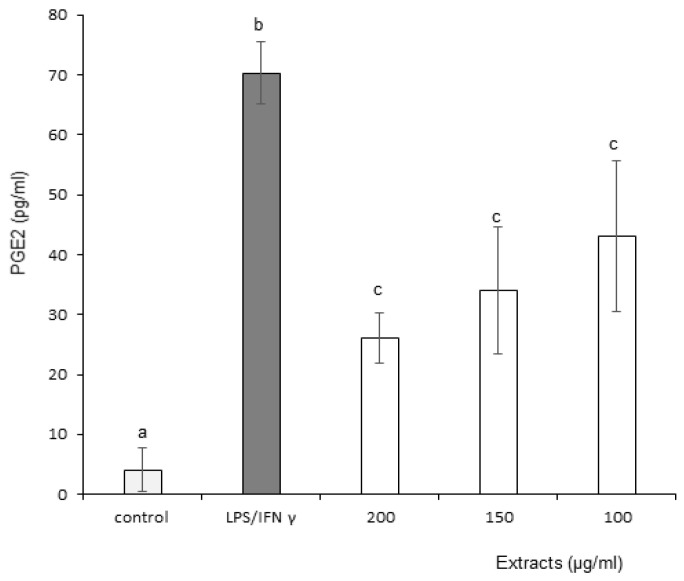
Effect of Ocimum basilicum stem ethanol extracts on Prostaglandin E2 (PGE-2) production. The values are expressed as mean ± SD (*n* = 3). The dissimilar letters on the graph represent significant differences (*p* < 0.05) according to one-way ANOVA followed by post hoc Tukey test. Error bars indicate ± SD (standard deviation).

Effects of OB extracts on Prostaglandin E2 (PGE-2) production are presented in Figure 9. The graph represents the level of PGE-2 production as a function of increasing concentrations of OB extracts. Leaf extracts did not generate a reduction in PGE-2 (data not shown). Conversely, stems decreased PGE-2 at only three concentrations: 100, 150 and 200 µg/mL. The graph suggests a concentration-dependent effect based on the numerical values (62.88 ± 6.67, 51.92 ± 12.10 and 38.62 ± 17.66 pg/mL, respectively), but it did not reach statistical significance. Therefore, stem extracts demonstrated a superior effect on PGE-2 inflammation marker.

### 3.4. Antispasmodic Activity

**Figure 10:**
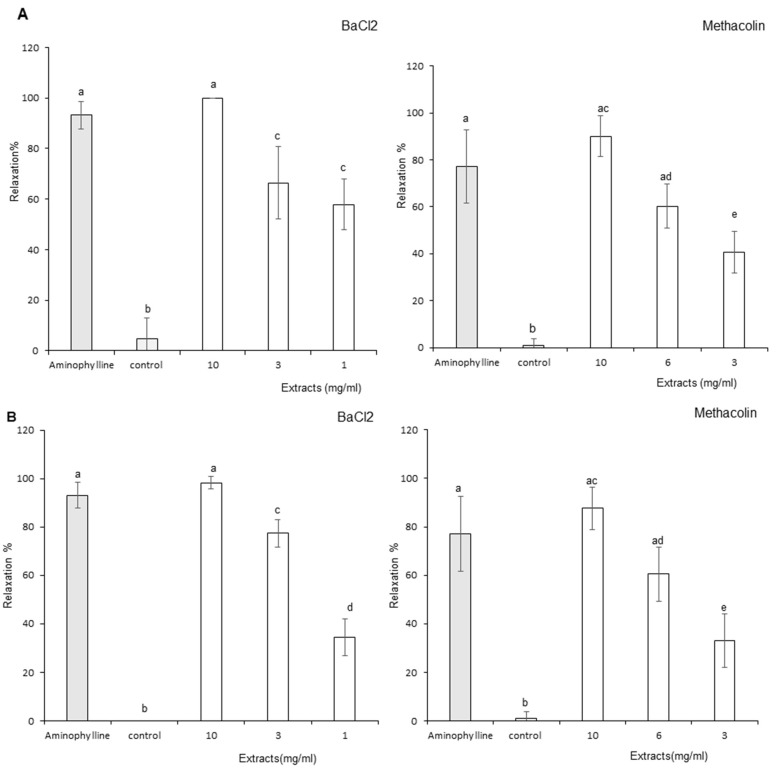
Effect of Ocimum basilicum extracts on jejunum rings stimulated by barium chloride (BaCl_2_) or Metacholin. (**A**) Leaf aqueous extract results, (**B**) stem aqueous extracts results. The dissimilar letters on the graph represent significant differences (*p* < 0.05) according to one-way ANOVA followed by post hoc Tukey test. Error bars indicate ± SEM (standard error to the mean).

The antispasmodic effects of leaf and stem extracts are presented in Figure 10A,B, respectively. BaCl_2_ and metacholine induced a sustained contraction on jejunum segments. Internal positive control aminophyline induced the expected relaxation.

Both leaf and stem aqueous extracts generated significant relaxation on BaCl_2_-induced contraction. Concentration-dependent relaxation was recorded for stems, but only partially for leaves. Relaxation generated by the lowest concentration of stem extracts was inferior when compared to the same concentration for leaf extracts.

Regarding metacholine-induced contraction, leaf and stem aqueous extracts generated significant relaxation. In both cases, relaxation was concentration dependent.

Therefore, both leaf and stem extracts demonstrated significant jejunum antispasmodic effects.

## 4. Discussion

*Ocimum basilicum* is recognized as an aromatic medicinal plant used in traditional medicine for a large panel of therapeutic indications, some of which have been confirmed by scientific studies [1]. Most studies have explored leaf aqueous or ethanol extracts as well as essential oil [31,42]. These extracts demonstrate antioxidant, anti-inflammatory, immunomodulatory, analgesic, anti-pyretic, anti-aging, anti-cancer and antispasmodic properties [43,44,45]. When exploring the potential bioactivities of vegetables, the choice of the plant subparts is known to influence the type and/or levels of bioactivity [21]. In this context, our study aimed at exploring the differential phytochemical and biological profile of two subparts of *Ocimum basilicum*, i.e., leaves and stems.

Leaf and stem extract compositions indicated the presence of phenolic acids, flavonoids, organic acids and fatty acids. Our results are in accordance with previous records from the literature about basil phytochemistry [31]. In addition, we observed differences in composition between leaves and stems. From a qualitative point of view, leaves contain flavonoids (salvigenin, rutin and gallocatechin), organic acid (ferroyl-tartaric acid) and phenolic acids (medioresinol) among others compounds. Stems contain flavonoids (vicenin, salvigenin), phenolic acids (rosmarinic acid, salvianolic acid) and fatty acid (stearic acid). From a quantitative point of view, the relative amounts also showed specificity for each sub-matrix respectively (leaves vs. stems), e.g., stearic acid (9% vs. 6%), salvigenin (5% vs. 6%), rutin (2% vs. nd, not detected), gallocatechin (1% vs. nd), rosmarinic acid (0% vs. 7%) or salvianolic acid (0% vs. 6%). These differences, on both qualitative and quantitative levels, between subparts of a vegetable have been reported in several plants. They may be related to specific functions and associated metabolic pathways of the considered part of the vegetable [20,21]. Furthermore, the pedoclimatic conditions of the growth of a plant [17], as well as the accession type of basil, may strongly influence its phytochemical profile and thereby the variations associated with the subparts of the plant. In addition, the extraction process, with either water or hydro-ethanol, also has an impact on the phytochemical profiles of the extracts [46]. The association of all these factors is of interest in terms of the relation between the plant’s phytochemistry and its bioactivity potential. Indeed, the constitutive compounds of the extracts, in addition to characterizing the vegetable and its subpart, define a specific combinations of bioactive and non-bioactive molecules, each possessing its own biological properties, potentially influencing the final resulting biological activity.

In this context, total polyphenol content (TPC) makes it possible to build a bridge between plant phytochemistry and bioactivity as a first step in evaluating the global putative antioxidant potential of the vegetable matrix. Our results indicate a higher TPC in leaf ethanol extract when compared to stems, but no difference was recorded between aqueous extracts. The levels of TPC are in the same range as previously reported for basil [46]. Polyphenols represent a large group of compounds including various chemical families, e.g., phenolic acids, flavonoids and tannins, recognized for their positive effects in various pathological conditions characterized by redox imbalance and inflammation [47]. The phytochemical profile and associated TPC are influenced by the vegetable subpart and extraction procedure, with leaves tending to have higher bioactive potential. This type of result was observed in basil when the authors compared leaves and flowers extracts [45]. Therefore, *Ocimum basilicum* leaves may bear a more pronounced antioxidant activity than other parts of the plant. Indeed phenolic compounds are secondary metabolites of plants with direct antioxidant effects as electrons donors, and in some case they may also stimulate cells’ endogenous antioxidant defense systems [48].

The DPPH assay mimics free radical production to assess the scavenging capacity of a test compound or extract based on its ability to provide a hydrogen atom. Basil antioxidant capacity evaluated by DPPH assay indicated that leaf and stem extracts have similar antioxidant activity in aqueous or ethanol solvent. However, the ethanolic procedure positively influenced the biological activity of the samples, with a higher antioxidant effect in both leaf and stem extracts when compared to aqueous ones, in accordance with the literature. The ORAC test monitors the scavenging of free radicals such as peroxyls, predominantly associated with lipid peroxidation. The ORAC assay data confirm the superiority of the antioxidant activity of leaf and stem ethanol extracts over the corresponding aqueous extracts. Nonetheless, in both solvent conditions, leaves appeared more active than stems. Taken together, the results from TPC, DPPH and ORAC assays suggest that leaf ethanol extracts have more potent antioxidant bioactivity potential than stem extracts. This difference in biological properties between the subparts of the plant and as a function of the extraction procedure have already been observed by other research groups in both basil (between leaves and flowers) [49] and other vegetables [50]. The phytochemical detected in both parts of the plant are known for their antioxidant potential. Indeed, compounds such as salvigenin, salvianolic acid, rosmarinic acid or vicenin and rutin are known for their health effects. They include antioxidant, anti-inflammatory, and also immunomodulation, anti-apoptotic and anti-tumorigenic properties [51]. Therefore, the difference in biological effects, in addition to accession type, growth conditions and extraction procedure, and without being exhaustive, may also rely on differential combinations of the quality and quantity of the compounds involved in a complex interplay of synergies and antagonisms [20].

Considering that leaf ethanol extracts were more bioactive (preliminary results, not shown), we focused our study on ethanol extracts of leaves and stems.

Inflammation processes and the associated inflammatory cytokine production are normal physiological non-specific immune responses when facing organism assault. In various metabolic diseases, such as inflammation-associated pathologies, obesity, cancer or cardiovascular diseases, they may play a pivotal role. In such cases, plant polyphenols and metabolites are known to bear in vitro and in vivo anti-inflammatory activities alleviating cells from the detrimental inflammation burden responsible for tissue damages [14]. The mechanism of action of polyphenols is proposed to modulate signaling pathways such as NF-kB (nuclear factor kappa-light-chain-enhancer of activated B cells), MAPk (mitogen-activated protein kinase), PI3K/Akt (Phosphatidylinositide 3-kinase/Protein kinase B), PLA2 enzyme (Phospholipase A2), cyclooxygenases (COXs) leading to reduction of inflammation markers production, i.e., Prostaglandin E2, Interleukin 6 or Monocytes Chemoattractant Protein-1. Furthermore, polyphenols are known to stimulate the activity of catalase, superoxide dismutase (SOD) and glutathione peroxidase, thereby improving endogenous anti-inflammatory defenses [52].

The anti-inflammatory properties of *Ocimum basilicum* (OB) were explored on stimulated macrophage cells in vitro through the measurement of inflammatory marker production. Nitric oxide (NO) is a free radical and an inflammation marker involved with various functions in different organs, i.e., vasodilation in the cardiovascular system or a mediator of the non-specific immune response [53]. Both extracts induced a concentration-dependent inhibition of nitric oxide (NO) production. In addition, the extracts also demonstrated the ability to quench NO free radicals in a similar concentration-dependent manner. These effects, associated with OB antioxidant capacity, corroborate the biological activity of stem extracts in oxidative stress conditions. This property might be explained by the combination of NO scavenging and an inhibitory effect of flavonoids on iNOS (inducible Nitric Oxide Synthase), as previously shown [52].

Conversely, TNF-α production was not affected by macrophage exposure to OB extracts. Neither leaves or stem extracts demonstrated a significant influence. This pro-inflammatory cytokine is known for its pleiotropic and contradictory effects through the modulation of inflammation onset and oncogenesis processes. Its beneficial influence seems to be associated with its concentration levels. Therefore, modulation of its production by bioactive compounds may be of variable interest as a function of the subject pathophysiological status [54]. Nonetheless, it is noteworthy to mention that basil ethanol extracts were demonstrated to have among the highest anti-tumor effects in the lamiaceae plant family. This anti-oncogenesis property and associated genoprotective effects would be mainly correlated with basil impact on oxidative stress and NO modulation rather than TNF. Therefore it might be suggested that the absence of variation of the latter cytokine in our assay, for both leaves and stems, might be considered as in accordance rather than as a discrepancy regarding the biological potential of Ocimum basilicum [49].

To further explore the anti-inflammatory and immunomodulatory potential of OB, we measured its influence on inflammation markers Interleukin 6 (IL-6), Monocyte Chemoattractant Protein-1 (MCP-1) and prostaglandin E2 (PGE-2) productions. Stem ethanol extracts clearly demonstrate (i) a concentration-dependent inhibition of the production of all three molecules and (ii) a statistically superior inhibition of the production of the three inflammation markers when compared to leaf ethanol extracts. These data suggest the potential interest of stem extracts for treating inflammation-associated diseases. The inhibition of PGE-2 production may be related to the observed reduction of IL-6 and MCP-1 production [55]. Phenolic acids and flavonoids are known to inhibit PLA2 (Phospholipase A2) and COX (Cycloxygenase) enzyme activities. This leads to reduction of prostaglandin production, which is associated with lower stimulation of IL-6 and MCP-1 production. This hypothesis may be compatible with the absence of effects of OB extracts on TNF-α and its signaling pathway. Indeed, TNF-α is associated with IL-6 production through the p42-44 MAPK pathway. A decrease in IL-6 and MCP-1 secretion could be generated via the inhibition of PLA2/COX pathway without significant effect on TNF-α/p42-44 MAPK signaling cascade [56,57,58,59]. Taken together, our results on inflammation demonstrate the anti-inflammatory properties of OB. They also unravel the specific more potent effects of stem extracts on inflammatory markers involved in the initiation and chemotaxis phase of inflammation with a possible prophylactic influence against the transition from acute to chronic condition. To our knowledge, this the first time that *Ocimum basilicum* stem extracts have been reported to demonstrate these bioactivities in vitro in comparison to classically used leaf extracts.

Among the various biological properties attributed to the Ocimum genus and its multiple species, spasmolytic properties have been claimed by a few reports for *Ocimum gratissimum* on rabbit jejunum, guinea pig ileum and rat vascular bed. These data were obtained with essential oils [57,58,59]. Regarding *Ocimum basilicum* aqueous extract, spasmolytic effect has only been explored on the responsiveness of isolated tracheal rings to methacholin [60].

Despite its use in traditional medicine for digestive troubles [1], the antispasmodic activity of *Ocimum basilicum* has never been tested on jejunum smooth muscle. We therefore investigated OB extract on this organ target. We used an aqueous extract, as ethanol caused tissue alteration and dysfunction. Smooth muscle contractions are present in various intestine pathologies with or without inflammation such as IBD (inflammatory bowel disease) or asthma, and they may be painful. Pharmacological agents able to inhibit these spams may contribute to alleviate both intestinal/tracheal disorder and pain [61]. BaCl_2_ induces calcium entry in the cell through a voltage-dependent calcium channel. This leads to hyperpolarization of the smooth muscle cell membrane and cause its contraction. Methacholine is a direct cholinergic agonist of muscarinic receptor inducing smooth muscle contraction. Both OB leaves and aqueous extracts demonstrated concentration-dependent relaxation of smooth muscle contraction induced by BaCl_2_ and Methacholine. These results clearly suggest that *Ocimum basilicum* stems and leaves have significant antispasmodic effects. This observation is in accordance with previous reports regarding the properties of Ocimum genus extracts on smooth muscles [57,58,59]. In addition, our data tend to indicate a dual mechanism of action for the observed antispasmodic effect that could be mediated by an anticholinergic action and a calcium channel blockade. This hypothesis is supported by previous results obtained on various Ocimum species and might be supported by the phytochemicals present in the extracts [28,60,61]. Further investigations are required to determine the precise pharmacological mechanism of action of OB extracts on smooth muscle contractility and to identify the compounds responsible for this action. Records from the literature suggest a direct effect, independent from autonomic central nervous system [28]. To our knowledge, it is the first time that *Ocimum basilicum* stem extracts have been reported to demonstrate this bioactivity ex vivo. It may contribute to scientifically confirming the traditional use of *Ocimum basilicum* in diseases with smooth vascular hyperactivity disorders such as IBD, asthma or hypertension.

## 5. Conclusions

In conclusion, the present study explored, for the first time, the antioxidant, anti-inflammatory and antispasmodic properties of *Ocimum basilicum* stem extracts.

Taken together, our results suggest several conclusions. First, OB stem extracts demonstrate biological activity of interest. Second, the subparts of the OB plant, i.e., stems and leaves, demonstrate differential phytochemistry that could be correlated with bioactivity variations, more notably with respect to anti-oxidant and anti-inflammatory properties, but not with respect to antispasmodic effects. Third, through processes, e.g., extraction, applied to a vegetable, it may be possible to selectively orient the physicochemical and thereby biological properties of an extract to target a given pathophysiological disturbance. In the present case, our data, combined with that found in the literature, argue for the potentially significant interest of *Ocimum basilicum* extracts, and more specifically stem extracts, for the prophylaxis and/or as adjuvant therapy in the treatment of intestinal relapsing diseases associated with inflammation such as IBD. Indeed, stems, in addition to their antispasmodic potential, have a marked impact on biomarkers involved in the initiating phases of inflammation and chemophylaxis, which may contribute to limit the acute phases of the pathology as well as pain through reduction of prostaglandin production and spasms intensity [28,61,62,63].

Finally, it should be mentioned that a high level of complexity emerges through the endeavor of understanding the nutrition-health potential of plant extracts. Future in-depth investigations are necessary to articulate the exploration of (i) the complex and intricated pathological regulatory pathways and associated mediators involved in the targeted diseases with (ii) the complex pharmacological interplay between bioactive and non-bioactive compounds present in the plant extracts. Our research group is engaged in unraveling these research questions.

## Figures and Tables

**Table 1:** Polyphenol profile in Ocimum basilicum leaf extract.

RT (min)	M/Z	Water	Ethanol	Identification Based on Bibliography	Ref
3.25	305.2	x	x	(+)-Gallocatechin	[32,33]
3.78	371.2	x	x	Medioresinole (Furanoide lignine)	[34,35]
4.31	503.3	x	x	luteolin acetyl-glucuronide	[35,36,37]
4.45	547.3	x	x	5.50-Dihydroxy-d-sesamin-5-O-glucoside	[38]
5.31	329.2	x		Trihydroxy octadecenoic acid (fatty Acid)	[34]
6.21	351.2		x	Salvigenin(5-Hydroxy-6,7,4′-trimethoxyflavone) (Flavon)	[34]
6.51	329.2	x	x	Trihydroxy octadecenoic acid (fatty Acid)	[34]
6.54	359.1		x	rosmarinic Acid	[34,35]
6.93	517.3	x		luteolin acetyl-glucuronide	[35,36,37]
7.07	345.1		x	Eupatorin (3′,5-Dihydroxy-4′,6,7-trimethoxyflavone) Flavon	[34]
8.05	329.1		x	Trihydroxy Acid octadecenoic ((Fatty Acid)	[34]
8.57	325.1	x	x	feruloyltartaric acid	[39,40,41]
9.62	325.1		x	feruloyltartaric acid	[39,41]
10.49	609.3		x	Rutin (flavonoid)	[34,36,37]

**Table 2:** Polyphenol profile in Ocimum basilicum stem extract.

RT (min)	M/Z	Water	Ethanol	Identification Based on Bibliography	Ref
1	191.1	x		Isocitric/citric acid	[34]
3.57	327.2	x		Salvigenin(5-Hydroxy-6,7,4′-trimethoxyflavone) Flavones	[34]
3.78	387.2	x	x	Medioresinole (Furanoide lignine)	[34,36]
4.21	507.2	x		Quercitin-acetyl-O-glucoside	[40]
4.32	503.3	x		luteolin acetyl-glucuronide	[35,36,37]
4.45	547.3	x	x	5.50-Dihydroxy-d-sesamin-5-O-glucoside	[38]
4.98	359.1		x	Rosmarinic acid	[34,35]
5.14	359.1		x	Rosmarinic acid	[34,35]
5.51	435.1	x		Naringenin glucoside	[41]
6.22	327.2	x	x	Salvigenin(5-Hydroxy-6,7,4′-trimethoxyflavone) Flavone	[34]
6.52	329.2	x	x	Trihydroxy octadecenoic acid (Fatty Acid)	[34]
7.85	311.2		x	Caftaric acid	[34,35,42]
8.06	329.1		x	Trihydroxy octadecenoic acid (fatty Acid)	[34]
8.33	359.1		x	Rosmarinic Acid	[34,35]
8.53	299.1		x	hydroxybenzoic acid-O-glucoside(salicylic acid-O-glucoside)	[39]
8.91	295.2		x	Cinnamyl malic acid	[42]
9.13	311.2	x	x	caftaric acid	[34,35]
9.74	353.2	x	x	Chlorogenic acid	[36,38]
10.48	609.3		x	Rutin (flavonoid)	[34,36,37]
10.63	593.3		x	Vicenin II (flavonoid)	[39]
10.77	593.3	x	x	feruloyltartaric acid	[39]

## Data Availability

Not applicable.

## References

[1] Bilal A., Jahan N., Ahmed A., Bilal S.N., Habib S., Hajra S. (2012). Phytochemical and pharmacological studies on *Ocimum basilicum* Linn-A review. Int. J. Curr. Res. Rev..

[2] Putievsky E., Galambosi B., Hiltunen R., Holm Y. (2005). Production Systems of Sweet Basil. Basil: The Genus Ocimum.

[3] Nadeem F., Hanif M.A., Bhatti I.A., Jilani M.I., Al-Yahyai R., Hanif M.A., Nawaz H., Khan M.M., Byrne H.J. (2020). Chapter 4: Basil. Medicinal Plants of South Asia.

[4] Pushpangadan P., George V., Peter K.V. (2012). Basil. Handbook of Herbs and Spices.

[5] Calderón Bravo H., Vera Céspedes N., Zura-Bravo L., Muñoz L.A. (2021). Basil Seeds as a Novel Food, Source of Nutrients and Functional Ingredients with Beneficial Properties: A Review. Foods.

[6] Naji-Tabasi S., Razavi S.M.A. (2017). Functional properties and applications of basil seed gum: An overview. Food Hydrocoll..

[7] Kisa D., İmamoğlu R., Genç N., Şahin S., Qayyum M.A., Elmastaş M. (2021). The interactive effect of aromatic amino acid composition on the accumulation of phenolic compounds and the expression of biosynthesis-related genes in *Ocimum basilicum*. Physiol. Mol. Biol. Plants.

[8] Irondi E.A., Agboola S.O., Oboh G., Boligon A.A. (2016). Inhibitory effect of leaves extracts of *Ocimum basilicum* and *Ocimum gratissimum* on two key enzymes involved in obesity and hypertension in vitro. J. Intercult. Ethnopharmacol..

[9] Al Abbasy D.W., Pathare N., Al-Sabahi J.N., Khan S.A. (2015). Chemical composition and antibacterial activity of essential oil isolated from Omani basil (*Ocimum basilicum* Linn.). Asian Pac. J. Trop. Dis..

[10] Vieira R.F., Simon J.E. (2000). Chemical Characterization of basil (*Ocimum* spp.) found in the markets and used in traditional medicine in Brazil. Econ. Bot..

[11] Abirami S.G., Nirmala P.A. (2014). Comparative in vitro study of anticancer effect of Mentha piperita, *Ocimum basilicum* and *Coleus aromaticus* against human laryngeal epidermoid carcinoma (HEP-2) cell lines. J. Med. Plants Stud..

[12] Javanmardi J., Khalighi A., Kashi A., Bais H.P., Vivanco J.M. (2002). Chemical Characterization of Basil (*Ocimum basilicuml* L.) Found in Local Accessions and Used in Traditional Medicines in Iran. J. Agric. Food Chem..

[13] Eftekhar N., Moghimi A., Mohammadian Roshan N., Saadat S., Boskabady M.H. (2019). Immunomodulatory and anti-inflammatory effects of hydro-ethanolic extract of *Ocimum basilicum* leaves and its effect on lung pathological changes in an ovalbumin-induced rat model of asthma. BMC Complement. Altern. Med..

[14] Egbuna C., Awuchi C.G., Kushwaha G., Rudrapal M., Patrick-Iwuanyanwu K.C., Singh O., Odoh U.E., Khan J., Jeevanandam J., Kumarasamy S. (2021). Bioactive Compounds Effective Against Type 2 Diabetes Mellitus: A Systematic Review. Curr. Top. Med. Chem..

[15] Bower A., Marquez S., De Mejia E.G. (2016). The Health Benefits of Selected Culinary Herbs and Spices Found in the Traditional Mediterranean Diet. Crit. Rev. Food Sci. Nutr..

[16] Da Costa A.S., de Arrigoni-Blank M.F., De Carvalho Filho J.L., De Santana A.D., de Santos D.A., Alves P.B., Blank A.F. (2015). Chemical diversity in basil (*Ocimum* sp.) germplasm. Sci. World J..

[17] Soro L.C., Munier S., Pelissier Y., Grosmaire L., Yada R., Kitts D., Ocho-Anin Atchibri A.L., Guzman C., Boudard F., Menut C. (2016). Influence of geography, seasons and pedology on chemical composition and anti-inflammatory activities of essential oils from Lippia multiflora Mold leaves. J. Ethnopharmacol..

[18] Ferrare K., Bidel L.P.R., Awwad A., Poucheret P., Cazals G., Lazennec F., Azay-Milhau J., Tournier M., Lajoix A.D., Tousch D. (2018). Increase in insulin sensitivity by the association of chicoric acid and chlorogenic acid contained in a natural chicoric acid extract (NCRAE) of chicory (*Cichorium intybus* L.) for an antidiabetic effect. J. Ethnopharmacol..

[19] Asgari Lajayer B., Najafi N., Moghiseh E., Mosaferi M., Hadian J. (2019). Effects of Gamma Irradiated and Non-Irradiated Sewage Sludge on Essential Oil Content and Constituents of *Ocimum basilicum* L. J. Med. Plants.

[20] Rui W., Xia W., Zhao W., Li B., Li J., Feng Y., Chen H., Zhao S. (2020). Differential Constituents in Roots, Stems and Leaves of *Polygonum multiflorum* Thunb. Screened by UPLC/ESI-Q-TOF-MS and Multivariate Statistical Analysis. J. Chromatogr. Sci..

[21] Wang J., Zhang J., Zhang C., Sun X., Liao X., Zheng W., Yin Q., Yang J., Mao D., Wang B. (2019). The qualitative and quantitative analyses of *Gelsemium elegans*. J. Pharm. Biomed. Anal..

[22] Genfi A.K.A., Larbie C., Emikpe B.O., Oyagbemi A.A., Firempong C.K., Adjei C.O. (2020). Modulation of Oxidative Stress and Inflammatory Cytokines as Therapeutic Mechanisms of *Ocimum americanum* L. Extract in Carbon Tetrachloride and Acetaminophen-Induced Toxicity in Rats. J. Evid. Based Integr. Med..

[23] Hakkim F.L., Shankar C.G., Girija S. (2007). Chemical composition and antioxidant property of holy basil (*Ocimum sanctum* L.) leaves, stems, and inflorescence and their in vitro callus cultures. J. Agric. Food Chem..

[24] Kelm M.A., Nair M.G., Strasburg G.M., DeWitt D.L. (2000). Antioxidant and cyclooxygenase inhibitory phenolic compounds from *Ocimum sanctum* Linn. Phytomedicine.

[25] Madeira S.V., Matos F.J., Leal-Cardoso J.H., Criddle D.N. (2002). Relaxant effects of the essential oil of *Ocimum gratissimum* on isolated ileum of the guinea pig. J. Ethnopharmacol..

[26] Madeira S.V., Rabelo M., Soares P.M., Souza E.P., Meireles A.V., Montenegro C., Lima R.F., Assreuy A.M., Criddle D.N. (2005). Temporal variation of chemical composition and relaxant action of the essential oil of Ocimum gratissimum L. (Labiatae) on guinea-pig ileum. Phytomedicine.

[27] Franca C.S., Menezes F.S., Costa L.C., Niculau E.S., Alves P.B., Pinto J.E., Marçal R.M. (2008). Analgesic and antidiarrheal properties of Ocimum selloi essential oil in mice. Fitoterapia.

[28] Souza S.D., Franca C.S., Niculau E.S., Costa L.C., Pinto J.E., Alves P.B., Marçal R.M. (2015). Antispasmodic effect of Ocimum selloi essential oil on the guinea-pig ileum. Nat. Prod. Res..

[29] Morel S., Arnould S., Vitou M., Boudard F., Guzman C., Poucheret P., Fons F., Rapior S. (2018). Antiproliferative and Antioxidant Activities of Wild Boletales Mushrooms from France. Int. J. Med. Mushrooms.

[30] Bony E., Boudard F., Dussossoy E., Portet K., Brat P., Giaimis J., Michel A. (2012). Chemical composition and anti-inflammatory properties of the unsaponifiable fraction from awara (*Astrocaryum vulgare* M.) pulp oil in activated J774 macrophages and in a mice model of endotoxic shock. Plant Foods Hum. Nutr..

[31] Deme P., Aluganti Narasimhulu C., Parthasarathy S. (2019). Evaluation of Anti-Inflammatory Properties of Herbal Aqueous Extracts and Their Chemical Characterization. J. Med. Food..

[32] Ma J., Yang H., Basile M.J., Kennelly E.J. (2004). Analysis of Polyphenolic Antioxidants from the Fruits of Three Pouteria Species by Selected Ion Monitoring Liquid Chromatography−Mass Spectrometry. J. Agric. Food Chem..

[33] Kumar S., Bouic P.J., Rosenkranz B. (2020). In Vitro Assessment of the Interaction Potential of *Ocimum Basilicum* (L.) Extracts on CYP2B6, 3A4, and Rifampicin Metabolism. Front. Pharmacol..

[34] Jayasinghe C., Gotoh N., Aoki T., Wada S. (2003). Phenolics Composition and Antioxidant Activity of Sweet Basil (*Ocimum Basilicum* L.). J. Agric. Food Chem..

[35] Ibrahim R.Y.M., Mansour S.M., Elkady W.M. (2020). Phytochemical Profile and Protective Effect of *Ocimum Basilicum* Aqueous Extract in Doxorubicin/Irradiation-Induced Testicular Injury. J. Pharm. Pharmacol..

[36] Lu Y., Gao B., Chen P., Charles D., Yu L. (2014). Characterisation of Organic and Conventional Sweet Basil Leaves Using Chromatographic and Flow-Injection Mass Spectrometric (FIMS) Fingerprints Combined with Principal Component Analysis. Food Chem..

[37] Ye M., Yan Y., Guo D. (2005). Characterization of Phenolic Compounds in the Chinese Herbal Drug Tu-Si-Zi by Liquid Chromatography Coupled to Electrospray Ionization Mass Spectrometry. Rapid Commun. Mass Spectrom..

[38] Farag M.A., Ezzat S.M., Salama M.M., Tadros M.G. (2016). Anti-Acetylcholinesterase Potential and Metabolome Classification of 4 Ocimum Species as Determined via UPLC/QTOF/MS and Chemometric Tools. J. Pharm. Biomed. Anal..

[39] Burducea M., Zheljazkov V.D., Lobiuc A., Pintilie C.A., Virgolici M., Silion M., Asandulesa M., Burducea I., Zamfirache M.-M. (2019). Biosolids Application Improves Mineral Composition and Phenolic Profile of Basil Cultivated on Eroded Soil. Sci. Hortic..

[40] Prinsi B., Morgutti S., Negrini N., Faoro F., Espen L. (2019). Insight into Composition of Bioactive Phenolic Compounds in Leaves and Flowers of Green and Purple Basil. Plants.

[41] Lee J., Scagel C.F. (2009). Chicoric Acid Found in Basil (*Ocimum Basilicum* L.) Leaves. Food Chem..

[42] Sestili P., Ismail T., Calcabrini C., Guescini M., Catanzaro E., Turrini E., Layla A., Akhtar S., Fimognari C. (2018). The potential effects of *Ocimum basilicum* on health: A review of pharmacological and toxicological studies. Expert Opin. Drug Metab. Toxicol..

[43] Selvakkumar C., Gayathri B., Vinaykumar K.S., Lakshmi B.S., Balakrishnan A. (2007). Potential anti-inflammatory properties of crude alcoholic extract of *Ocimum basilicum* L. in human peripheral blood mononuclear cells. J. Health Sci..

[44] Singh S. (1999). Mechanism of action of anti-inflammatory effect of fixed oil of *Ocimum basilicum* Linn. Indian J. Exp. Biol..

[45] Noor Z.I., Ahmed D., Rehman H.M., Qamar M.T., Froeyen M., Ahmad S., Mirza M.U. (2019). In Vitro Antidiabetic, Anti-Obesity and Antioxidant Analysis of *Ocimum basilicum* Aerial Biomass and in Silico Molecular Docking Simulations with Alpha-Amylase and Lipase Enzymes. Biology.

[46] Ghasemzadeh A., Ashkani S., Baghdadi A., Pazoki A., Jaafar H.Z., Rahmat A. (2016). Improvement in Flavonoids and Phenolic Acids Production and Pharmaceutical Quality of Sweet Basil (*Ocimum basilicum* L.) by Ultraviolet-B Irradiation. Molecules.

[47] Amiot M.J., Riva C., Vinet A. (2016). Effects of dietary polyphenols on metabolic syndrome features in humans: A systematic review. Obes. Rev..

[48] Ninfali P. (2003). Polyphenols and antioxidant capacity of vegetables under fresh and frozen conditions. J. Agric. Food Chem..

[49] Oalđe Pavlović M., Kolarević S., Đorđević J., Jovanović Marić J., Lunić T., Mandić M., Kračun Kolarević M., Živković J., Alimpić Aradski A., Marin P.D. (2021). A Study of Phytochemistry, Genoprotective Activity, and Antitumor Effects of Extracts of the Selected Lamiaceae Species. Plants.

[50] Sevgi E., Dag A., Kızılarslan-Hançer Ç., Atasoy S., Kurt B.Z., Aksakal Ö. (2021). Evaluation of cytotoxic and antioxidant potential of *Dittrichia viscosa* (L.) Greuter used in traditional medicine. J. Ethnopharmacol..

[51] Fraga C.G., Croft K.D., Kennedy D.O., Tomás-Barberán F.A. (2019). The effects of polyphenols and other bioactives on human health. Food Funct..

[52] Yahfoufi N., Alsadi N., Jambi M., Matar C. (2018). The Immunomodulatory and Anti-Inflammatory Role of Polyphenols. Nutrients.

[53] Incalza M.A., D’Oria R., Natalicchio A., Perrini S., Laviola L., Giorgino F. (2018). Oxidative stress and reactive oxygen species in endothelial dysfunction associated with cardiovascular and metabolic diseases. Vasc. Pharmacol..

[54] Mercogliano M.F., Bruni S., Mauro F., Elizalde P.V., Schillaci R. (2021). Harnessing Tumor Necrosis Factor Alpha to Achieve Effective Cancer Immunotherapy. Cancers.

[55] Meneses C.C.B., Pizzatto L.N., Andrade F.F., Sipert C.R. (2020). Prostaglandin E_2_ Affects Interleukin 6 and Monocyte Chemoattractant Protein 1/CCL2 Production by Cultured Stem Cells of Apical Papilla. J. Endod..

[56] Gomes A., Fernandes E., Lima J.L., Mira L., Corvo M.L. (2008). Molecular mechanisms of anti-inflammatory activity mediated by flavonoids. Curr. Med. Chem..

[57] Aziba P.I., Bass D., Elegbe Y. (1999). Pharmacological investigation of *Ocimum gratissimum* in rodents. Phytother. Res..

[58] Pires A.F., Madeira S.V., Soares P.M., Montenegro C.M., Souza E.P., Resende A.C., de Soares Moura R., Assreuy A.M., Criddle D.N. (2012). The role of endothelium in the vasorelaxant effects of the essential oil of *Ocimum gratissimum* in aorta and mesenteric vascular bed of rats. Can. J. Physiol. Pharmacol..

[59] Interaminense L.F., Jucá D.M., Magalhães P.J., Leal-Cardoso J.H., Duarte G.P., Lahlou S. (2007). Pharmacological evidence of calcium-channel blockade by essential oil of *Ocimum gratissimum* and its main constituent, eugenol, in isolated aortic rings from DOCA-salt hypertensive rats. Fundam. Clin. Pharmacol..

[60] Eftekhar N., Moghimi A., Hossein Boskabady M., Kaveh M., Shakeri F. (2019). *Ocimum basilicum* affects tracheal responsiveness, lung inflammatory cells and oxidant-antioxidant biomarkers in sensitized rats. Drug Chem. Toxicol..

[61] Makharia G.K. (2011). Understanding and treating abdominal pain and spasms in organic gastrointestinal diseases: Inflammatory bowel disease and biliary diseases. J. Clin. Gastroenterol..

[62] Lahlou S., Interaminense L.F., Leal-Cardoso J.H., Morais S.M., Duarte G.P. (2004). Cardiovascular effects of the essential oil *of Ocimum gratissimum* leaves in rats: Role of the autonomic nervous system. Clin. Exp. Pharmacol. Physiol..

[63] Rashidian A., Roohi P., Mehrzadi S., Ghannadi A.R., Minaiyan M. (2016). Protective Effect of *Ocimum basilicum* Essential Oil Against Acetic Acid-Induced Colitis in Rats. J. Evid. Based Complement. Altern. Med..

